# 

**Published:** 1860-04-01

**Authors:** 


### CONTENTS.

| ART. | | PAGE |
|---|---|---|
| | Psychological Quarterly Retrospect | xix—xl |
| I. | On Habits of Intoxication as Causing a Type of Disease | 125 |
| II. | The Platonic Dialogues | 144 |
| III. | The Asylums of Spain | 162 |
| IV. | The Mental Philosophy of Fichte the Younger | 175 |
| V. | Pinel: a Biographical Study | 184 |
| VI. | Bain's Psychology | 205 |
| VII. | Nervousness | 218 |
| VIII. | Dr. B. A. Morel on Mental Disorders | 233 |
| IX. | On Epilepsy | 241 |
| X. | Modern Magicians and Mediomaniacs | 246 |
| | Foreign Psychological Literature | 261 |

